# Hygiene and disinfection measures for monkeypox virus infections

**DOI:** 10.3205/dgkh000421

**Published:** 2022-10-17

**Authors:** Maren Eggers, Martin Exner, Jürgen Gebel, Carola Ilschner, Holger F. Rabenau, Ingeborg Schwebke

**Affiliations:** 1 Association for Applied Hygiene e.V. (VAH), Bonn, Germany; 2 Society of Virology (GfV), Heidelberg, Germany; 3 Disinfectant Commission of the German Association for the Control of Virus Diseases e.V. (DVV), Kiel, Germany; 4 Labor Prof. Gisela Enders MVZ GbR, Stuttgart, Germany; 5 German Society of Hospital Hygiene (DGKH), Berlin, Germany; 6 University Hospital Bonn, Bonn, Germany; 7 University Hospital Frankfurt, Germany

**Keywords:** monkeypox, infection prevention and control, disinfection, hygiene, health care

## Abstract

In Germany, recommendations on infection prevention and control of current virus outbreaks are given as communications by the Association for Applied Hygiene e.V. (VAH) together with the joint Disinfectant Commission of the German Association for the Control of Virus Diseases e.V. (DVV) and the Society of Virology* (GfV). The DVV was founded in 1954 in response to the ongoing threat to the population from polio and was given its current name in 1977. The DVV is supported by the Federal Ministry of Health, the Ministries of Health of the Federal States, scientific societies, as well as social foundations and organisations. Private individuals cannot be members of the DVV. The Society of Virology e.V. (GfV) is a scientific society for all virological fields in Germany, Austria and Switzerland, and is thus the largest virological society in Europe. With numerous commissions, guidelines and statements, it is the authoritative contact for research, healthcare and politics. The joint commission “Virus Disinfection” of these scientific societies focuses on the efficacy of chemical disinfection procedures against viruses. The VAH bundles the expertise of scientific societies and experts on infection prevention and is particularly committed to the quality assurance of hygiene measures. With the VAH disinfectant list, the association provides the standard reference for the selection of high-quality disinfection procedures. This disinfectant list has a tradition of more than 60 years in Germany.

The original German version of this document was published in August 2022 and has now been made available to the international professional public in English. The document contains recommendations on hygiene and disinfection measures for monkeypox virus infections. Disinfectants against monkeypox must have at least proven efficacy against enveloped viruses (active against enveloped viruses); products with the efficacy ranges “limited virucidal activity” and “virucidal” can also be used. The disinfectant list of the VAH or the disinfectant list of the Robert Koch Institute are available for the selection of products. Especially in the case of contamination with crust or scab material, it should be noted that protein contamination can have a protective or stabilising effect on monkeypox. Therefore, cleaning – before disinfection – should always be carried out in this situation. Preventive measures such as vaccination and hygiene in the vicinity of people with monkeypox must be taken to prevent transmission to small children, pregnant women or people with a pronounced immune deficiency.

## Abstract (de)

In Deutschland geben der Verbund für angewandte Hygiene e.V. (VAH) zusammen mit der Kommission „Virusdesinfektion“ der Deutschen Vereinigung zur Bekämpfung der Viruskrankheiten e.V. (DVV) und der Gesellschaft für Virologie e.V. (GfV) Mitteilungen und Empfehlungen zu durch Viren übertragbare Krankheiten heraus. Der Schwerpunkt liegt dabei auf wirksamen Hygiene- und Desinfektionsmaßnahmen zur Prävention und Kontrolle bei gehäuftem Auftreten von Virusinfektionen. Die DVV wurde 1954 als Reaktion auf die andauernde Gefährdung der Bevölkerung durch die Poliomyelitis gegründet und erhielt 1977 ihren heutigen Namen. Die DVV wird vom Bundesministerium für Gesundheit, den Gesundheitsministerien der Bundesländer, wissenschaftlichen Fachgesellschaften sowie sozial engagierten Stiftungen und Organisationen getragen. Einzelpersonen können nicht Mitglied der DVV sein. Die Gesellschaft für Virologie e.V. (GfV) ist eine Fachgesellschaft für alle virologischen Fachgebiete in Deutschland, Österreich und der Schweiz und damit die größte virologische Fachgesellschaft in Europa. Mit zahlreichen Kommissionen, Leitlinien und Stellungnahmen ist sie zu virologischen Themen der maßgebende Ansprechpartner für Forschung, Gesundheitswesen und Politik. Die gemeinsame Kommission „Virusdesinfektion“ dieser Fachgesellschaften nimmt die Wirksamkeit chemischer Desinfektionsverfahren gegenüber Viren in den Fokus. Der VAH bündelt die Expertise von Fachgesellschaften und Fachleuten zur Infektionsprävention und setzt sich insbesondere für die Qualitätssicherung von Hygienemaßnahmen ein. Mit der Desinfektionsmittel-Liste des VAH gibt der Verbund die Standardreferenz zur Auswahl von qualitativ hochwertigen Desinfektionsverfahren heraus. Diese Desinfektionsmittel-Liste hat in Deutschland eine mehr als 60jährige Tradition.

Die deutsche Originalfassung des vorliegenden Übersichtsartikels zur aktuellen Situation der Affenpocken wurde im August 2022 veröffentlicht und wird jetzt auf Englisch der internationalen Fachöffentlichkeit zur Verfügung gestellt. Der Artikel enthält Empfehlungen zu Hygiene- und Desinfektionsmaßnahmen bei Infektionen mit Affenpocken-Viren. Desinfektionsmittel gegen Affenpocken-Viren müssen mindestens eine nachgewiesene Wirksamkeit gegen behüllte Viren

(„begrenzt viruzid“) aufweisen; Produkte mit den Wirkbereichen „begrenzt viruzid PLUS“ und „viruzid“ können ebenfalls verwendet werden. Zur Auswahl von Produkten stehen die Desinfektionsmittel-Liste des VAH oder die Desinfektionsmittel-Liste des Robert Koch-Instituts zur Verfügung. Besonders bei Verunreinigungen mit Krusten- oder Schorfmaterial ist zu beachten, dass die Proteinbelastung eine schützende bzw. stabilisierende Wirkung auf Affenpocken haben kann. Daher ist hier stets eine gründliche Reinigung – vor der Desinfektion – durchzuführen. Durch Präventivmaßnahmen wie Impfungen und Hygieneverhalten gilt es, im Umfeld von an Affenpocken erkrankten Personen, Übertragungen auf Kleinkinder, Schwangere oder Personen mit einer ausgeprägten Immundefizienz zu verhindern.

## 1 Introduction

**Figure 1:**
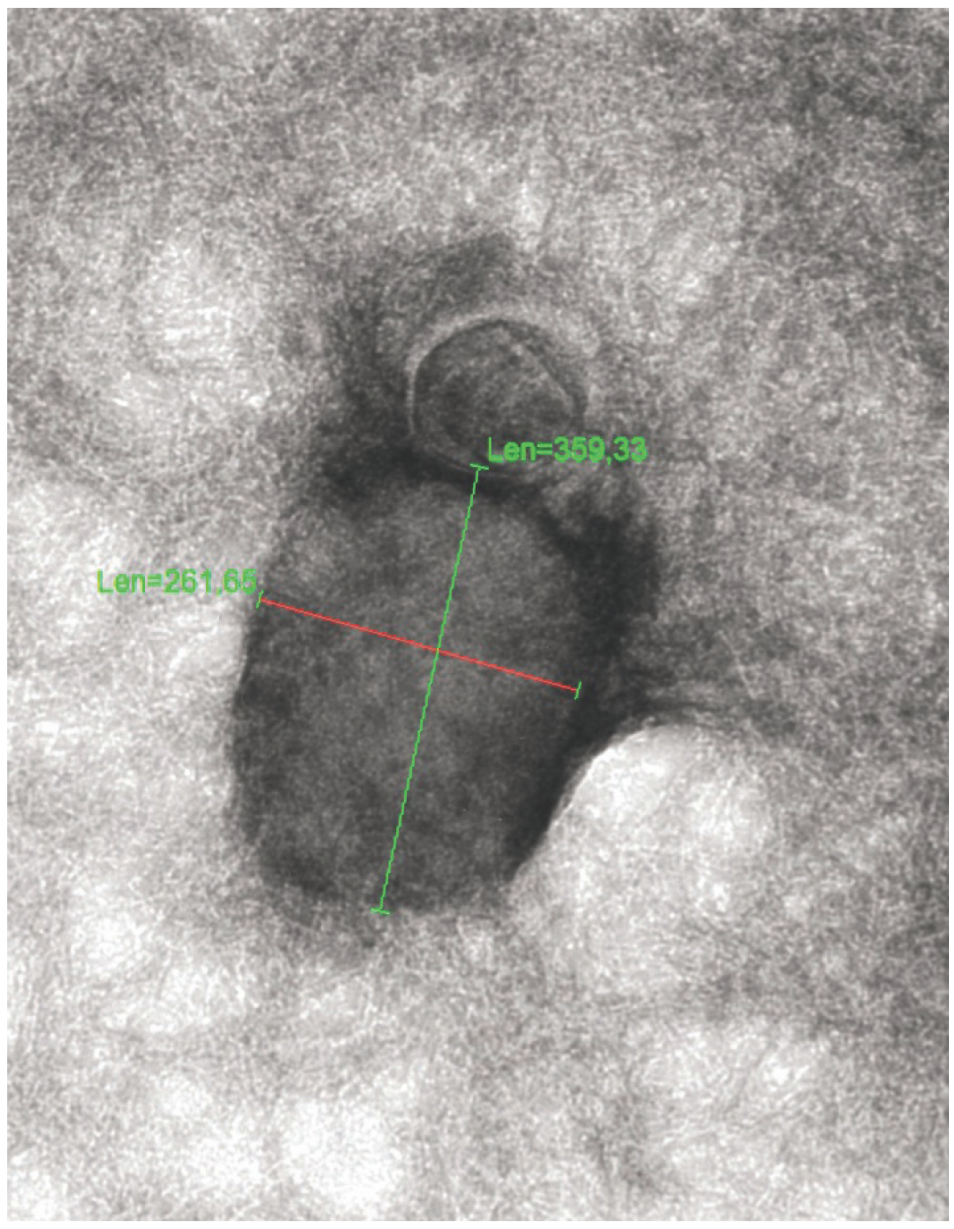
Electron micrograph of a monkeypox virus; magnification: 50,000x; size: approx. 360x240 nm; photo: © Prof. Holger F. Rabenau, Frankfurt University Hospital

Monkeypox is a zoonotic viral disease caused by infection with the monkeypox virus. This is an enveloped double-stranded DNA virus and belongs to the genus of orthopoxviruses of the *Poxviridae* family. The genus of orthopoxviruses also includes, among others, the variola virus (causative agent of smallpox), the vaccinia virus, the horsepox virus and the cowpox virus (Figure 1 (Fig. 1)). 

At the end of the 18^th^ century, the English doctor Edward Jenner used cowpox lymph (vaccina, derived from vacca, the cow) as a vaccine for the first time. The procedure called "vaccination" quickly became established. In addition to the variolation of cows or human-to-human vaccination, other animals such as rabbits, pigs, sheep, donkeys, horses and goats were also used as intermediate hosts to improve the effectiveness of the lymph. In fact, vaccines derived from cowpox or horsepox were used interchangeably for smallpox vaccination in the 19^th^ century [1]. The vaccinia virus, whose origin is not entirely clear due to the different intermediate hosts and of which there are four standardised variants, is still used today in a modified form for protection against smallpox (variola virus, monkeypox virus) as well as in research, e.g., as a test virus for testing the efficacy of disinfectants [2]. 

Monkeypox was first discovered in 1958, when two outbreaks of a smallpox-like disease occurred in (macaque) monkey colonies kept for research purposes. This is where the name “monkeypox” originates. The natural reservoir of monkeypox viruses is still unknown, but they have a broad host range. Antibodies against monkeypox have been detected in various rodent species (red squirrels, sun squirrels, Gambian giant hamster rats and other mouse and rat species), but also in mongooses, guenons and marmosets [3]. The first case of monkeypox in humans was detected in the Democratic Republic of Congo (DRC) in 1970, when efforts to eradicate smallpox were intensified. Since then, monkeypox in humans has been described in several other Central and West African countries: Cameroon, Central African Republic, Côte d’Ivoire, DRC, Gabon, Liberia, Nigeria, Republic of Congo and Sierra Leone [3], [4]. An increase in cases has been observed since 2017 [5]. 

Based on sequence analyses of isolates of monkeypox virus, these have so far been divided into two clades (variants): the West African and the Central African viruses. The latter occur mainly in the Congo Basin. Compared to the West African virus strains, which usually cause milder infections, they lead to infections with higher lethality (case fatality rate [CFR]%, i.e., the proportion of fatal courses of a disease: approx. 11 vs. 1%), more severe courses of disease and higher reproductive numbers (R0: 0.8 vs. 0.3). These data come from Africa, and mostly children are affected [3]. 

Outside Africa, human cases of monkeypox associated with international travel or animal imports have been rare. In 2003, the first major monkeypox outbreak occurred in the U.S. due to the transmission of the virus from infected prairie dogs. The prairie dogs had contracted the virus from animals imported from Gambia with whom they housed in an enclosure. Transmission to humans occurred via both invasive (bites, scratches) and non-invasive contacts (e.g., touching, feeding) [6]. Individual travel-associated case reports have been reported, for instance, from Singapore [7] as well as the US, Israel and the UK [8], [9].

## 2 Epidemiology of the current outbreak

Since May 6, 2022, 15,734 travel-independent confirmed cases have been reported worldwide (Global Health Mapbox, https://map.monkeypox.global.health/country, as of 22 July 2022). This 2022 outbreak cluster belongs to a new clade 3 identified by genome sequencing, which also includes clade 2 of the West African variant [10]. 

According to the Pan American Health Organizsation, between January 1 and July 7, 2022, a total of 7,892 confirmed cases were reported (from 63 Member States in 5 of the WHO regions), including 3 deaths (from Nigeria and the Central African Republic). As of July 7, 2022, 82% (6,496 cases in 34 countries) of confirmed cases have been reported in the WHO European Region; 15% (1,184 cases in 14 countries) in the WHO American Region; 2% (173 cases in 8 countries) in the WHO African Region; 1% (24 cases in 4 countries) in the WHO Western Pacific Region; and <1% (15 cases in 3 countries) in the WHO Eastern Mediterranean Region. In the last 7 days [2* to 9 July 2022*], there has been a 41.6% increase in reported cases globally. In the same period, there was an 82% increase in the Africa Region, 57% in the Americas Region, and 38% in the Europe Region. Thus, it is a very dynamic event. Globally, 78% of confirmed cases are men aged 18 to 44 years. Overall 98% of cases were identified as men who have sex with men (MSM), and of these, 41% are HIV-positive. Of the cases, 47% reported prior exposure to the disease during social events involving sexual contact. Of the 1,110 cases for which information is available, 113 are healthcare workers. Whether the infection in these cases was caused by occupational exposure is currently being investigated [11].

In Germany, cases have been reported from all federal states [12], [13]; the obligation to report confirmed cases according to §6 and 7 of the Infection Protection Act (IfSG) applies (1,924 cases as of 18 July 2022). Even though the WHO declared the outbreak a “public health emergency of international concern” on 23 July 2022 due to its dynamics, the Robert Koch Institute (as of 25 July 2022) continues to estimate the risk to the general population as low [12].

The WHO has established an Emergency Committee and published recommendations for action to protect public health. It also reported cases of infected children in the United Kingdom, in Spain and France with mild courses [14]. 

## 3 Clinical symptomatology

Monkeypox is a rare but potentially serious viral disease that typically begins after an incubation period of 5–21 days with a flu-like illness (initially with fever (>38.3°C), headache, muscle pain and fatigue) and swelling of the lymph nodes (cervical, inguinal) and develops into a rash on the face and body.

The eruptive stage begins with typical enanthema (oropharynx) and exanthema on the face, hands, forearms with centripetal spread over the body and subsequent development of redness and pox-typical uniform efflorescence stages (macules, vesicles, pustules and crusts). This occurs in about 80% of patients within a few days – 20% of sufferers develop polymorphous exanthema– similar to varicella. The lesions heal after drying and desquamation (sometimes with scarring). It was previously assumed that the infectivity lasts from the beginning of the prodromal stage at least until the crust of the skin lesions falls off [15]. 

People vaccinated against smallpox generally develop fewer efflorescences than non-vaccinated persons. In addition, ulcerations on the mucous membranes of the oral cavity with pharyngitis and tonsillitis, conjunctivitis with eyelid oedema and very painful lesions in the genital area frequently appear in non-vaccinated persons. Rarely, blindness and disfiguring scars occur as permanent damage. Severe, fatal haemorrhagic forms are rare; mild forms with less than 10 pockmarks and subclinical infections are sometimes observed.

Overall, the prognosis can be considered favourable. A higher probability of severe disease and mortality has only been observed in children under 8 years of age in the past [16], [17]. 

### 3.1 Clinical symptomatology: specific features of the monkeypox outbreak in 2022 (clade 3)

Recent reports show that the symptomatology of the monkeypox clade 3 diseases now prevalent in Europe differs from earlier descriptions. Symptoms of the prodromal stage are often absent. The skin lesions first appear in the urogenital and anal regions and not on the hands and soles of the feet, as is more common. The lesions were also often at different stages and may have occurred before systemic symptoms. The team of authors of a study published in Lancet Infectious Diseases in July 2022 therefore suggest an adjustment of the case definition [18].

Asymptomatic courses are also reported in a preprint study from Belgium. In anorectal swabs, a positive result could be detected in three (1.3%) of 224 MSM retrospectively tested for monkeypox. All those affected stated that they had not had any symptoms [19].

The possibility of generalised and severe courses in the case of impaired immunity (e.g., infants and young children, patients with immunosuppressive treatment, patients with chronic immunodeficiency, elderly people, pregnant women) should be taken into account. Therefore, it is also important to provide special protection for these population groups.

When reporting vaccination status, it is advisable to record the vaccination status from the vaccination record. A study by Eurosurveillance found that positive vaccination status against smallpox virus was also reported by patients under 40 [20].

## 4 Transmission routes

Monkeypox virus can be transmitted via different routes, namely animal-to-animal (predominantly different rodent species), animal-to-human, and human-to-human. In addition, the possibility of human-to-animal transmission cannot be ruled out in the case of contact with high virus loads. Monkeys as well as humans are false hosts for the monkeypox virus [21]. 

### 4.1 Human-to-human transmission

In human-to-human transmission in the current outbreak, the focus is on direct transmission via close contact with infectious skin lesions (e.g. via ruptured blisters). The blisters and pustules contain high viral loads. Ports of entry are small skin lesions as well as the mucous membranes and the respiratory tract. 

In addition, indirect transmission via infectious material (e.g. via bed linen [skin/scab particles], towels, clothing, hand contact surfaces) is possible. Vertical transmission from mother to child has also been described in rare cases [22], [23]. Transmission via larger respiratory droplets after prolonged personal contact seems to play a rather minor role in this outbreak, but may become relevant for prevention measures in the context of major events.

In a hospital in Hamburg, a systematic investigation of the viral load on selected surfaces of two patient rooms with anterooms was carried out on the 4^th^ day of accommodation of monkeypox patients. By means of PCR analysis, it was found that the surfaces of the wet cells close to the patients (water tap, soap dispenser lever, toilet seat) had a particularly high viral load, as did seating surfaces of chairs and the display of the patients’ mobile phones, as well as textiles (pillows, clothing around the anal region) used by the patients. Furthermore, surfaces that were presumably touched by medical staff and thus contaminated, such as cupboard handles, door handles of the anteroom, showed high viral loads. The authors restrictively point out that these are mainly results of a PCR analysis, i.e. viral DNA, and not the cultivation of infectious monkeypox viruses. Interestingly, however, they were able to culture monkeypox virus in three of the samples collected from one patient, namely from the investigator's glove, the operating lever of the soap dispenser and a towel on the patient's bed. All three samples had more than 10^6^ copies per sample (>10^3^ cp/cm^2^) [24].

The transmission of monkeypox virus via semen, urine, stool, blood and tear fluid has also not yet been conclusively clarified, although positive PCR test results appeared in some of these materials in a recent study [17]. The evidence thickens in an even more recent Spanish study from Barcelona, which shows how frequently the virus is found not only in skin lesions, but also in the throat, urine and semen. The Robert Koch Institute succeeded in cultivating replicable viruses from ejaculate [25]. PCR-positive results were also obtained in stool [26]. 

The question of air transferability or drift has also not yet been clarified. Therefore, window ventilation should only take place with the door closed (no cross-ventilation).

The longest chains of infection observed so far involved six to nine people [27].

## 5 Characteristics of monkeypox

Investigations with the vaccinia virus – related to the monkeypox virus – showed that this virus can remain infectious on surfaces for up to 56 days [28]. Stability on textile fibres was also investigated with the vaccinia virus. According to this, the virus could still be cultivated from wool fabric after up to four weeks and from cotton after four to eight days; textiles contaminated with virus-containing dust even remained infectious for up to twelve weeks [29], [30]. The publication by Adler et al. indicates that in some patients the virus could be detected in the throat swab by PCR test for up to three weeks (in one case from 2018 even up to 41 days) after diagnosis [17]. Whether this was only “residual nucleic acid” or infectious virus was not investigated.

The period during which a human being infected with monkeypox is infectious is currently estimated to be up to 4 weeks. The infectious dose of monkeypox virus is not known. In non-human primates, infection could be induced by intrabronchial administration of 5×10^4^ plaque-forming units (PFU), i.e. approx. 50,000 viruses [31] 

According to current knowledge, the environmental stability is comparable to that of the vaccinia virus. Monkeypox viruses are very resistant to desiccation and can survive in the crusts of skin lesions for months to years [32].

## 6 Prevention measures

### 6.1 Vaccination

In 1980, human smallpox was declared eradicated worldwide and vaccinations against smallpox were discontinued in 1976 in the then FRG and in 1982 in the GDR. Subsequently, the monkeypox virus spread as the most important smallpox virus – apart from cowpox (which was transmitted e.g. via “cuddly rats” kept as pets) – for public health [33], [34], [35]. 

In the EU, the smallpox vaccine Imvanex is licensed against smallpox, which contains a modified form of the vaccinia virus Ankara (MVA) that is no longer able to replicate. In the U.S. and Canada, the approval of this vaccine also extends to vaccination against monkeypox. In the European Medicines Agency (EMA), the review of data on the indication extension of Imvanex has started [36]. According to the recommendation of the Standing Committee on Vaccination (STIKO) of 21.6.2022, vaccination with Imvanex (MVA-BN) is currently recommended under certain conditions for post-exposure prophylaxis after monkeypox exposure of asymptomatic persons and as an indication vaccination of persons with an increased risk of exposure and infection [37].

In the meantime, the vaccine is available in the practices in Germany, and vaccinations according to the STIKO recommendations have started. The organisation and vaccination is regulated by the federal states.

### 6.2 Hygiene measures

The most important non-pharmaceutical preventive measure for the further spread and disease of monkeypox is the avoidance of close contact with an infected person. Patients and also the persons living in the same household with a monkeypox patient should be advised by a doctor and, if possible, trained on which hygiene measures to take and how to carry them out properly (Table 1 (Tab. 1) and Table 2 (Tab. 2)).

#### 6.2.1 Disinfection

Smallpox viruses are enveloped viruses that can be inactivated by disinfectants with proven **“virucidal activity against enveloped viruses”** [38]. As against SARS-CoV-2, disinfectants with a proven **“virucidal activity against enveloped viruses”** efficacy are in principle suitable for disinfection. In comparative studies with various enveloped viruses (e.g., hepatitis C virus, Ebola virus, influenza virus, coronavirus), the European test virus vaccinia virus proved to be the most resistant virus [38], [39], [40], [41], [42], [43]. The environmental stability of vaccinia viruses and monkeypox viruses is comparable [44], [45]. 

Products with the active ranges **“limited spectrum of virucidal activity”** and “virucidal activity” can also be used [38]. The disinfectant list of the VAH or the disinfectant list of the Robert Koch Institute are available for the selection of products. 

With regard to the problem of the stability of the virus, e.g., in skin flakes and crusts, it is important to ensure that the efficacy testing of surface disinfectants under high organic load has been carried out in accordance with the applicable test standards in the practical test in accordance with the requirements and methods for VAH certification of chemical disinfection procedures Annex V [46], [47]. Visibly contaminated near-patient surfaces with skin flakes and skin crusts should be removed in advance with a disposable disinfection wipe, which is then immediately disposed of in the residual waste. Disposable gloves must be worn for all cleaning and disinfection procedures, and hand disinfection must be performed after their use.

The use of disinfectants with the effective range **“limited spectrum of virucidal activity” or “virucidal activity”** would only be discussed (e.g., for surface disinfection) if, due to their mechanism of action, they could penetrate crusts/scabs and also inactivate the virus inside, if prior efficient cleaning of the surfaces is not possible. 

Laundry disinfectants are an exception here, as the European standardisation and also the VAH and RKI lists only specify the virucidal range of action. 

If no VAH certificate is available, it is recommended that an evaluation of the test reports and expert opinions submitted by the manufacturer be carried out by independent experts [48], [49]. 

The criterion for the selection of a disinfectant should not be a specific active ingredient or group of active ingredients, but the manufacturer-independent proof of efficacy for the required spectrum of activity for a specific product. 

The importance of laundry preparation, including the preparation of mopping utensils, for instance, for floor cleaning (mops), should be emphasised. Care must be taken that bed linen and body laundry is collected in such a way that, as far as possible, there is no environmental contamination with skin crusts, as the viruses embedded in these are much more difficult for disinfectants to reach. Chemo-thermal reprocessing with a virus-effective, VAH- or RKI-listed procedure is required for laundry reprocessing in the clinical and nursing environment [50]. 

## 7 Conclusion

Disinfectants against monkeypox must have at least proven efficacy against enveloped viruses (“active against enveloped viruses”); products with the efficacy ranges “limited virucidal activity” and “virucidal” can also be used. The disinfectant list of the VAH or also the disinfectant list of the Robert Koch Institute are available for the selection of products [51]. Especially in the case of contamination with crust or scab material, it should be noted that protein contamination can have a protective or stabilising effect on monkeypox. Therefore, cleaning before disinfection should always be carried out in this situation. Preventive measures such as vaccination and hygiene in the vicinity of persons with monkeypox must be taken to prevent transmission to small children, pregnant women or persons with a pronounced immune deficiency.

## Notes

### Competing interests

The authors declare that they have no competing interests.

### Citation reference:

This recommendation is also available in German:

Eggers M, Exner M, Gebel J, Ilschner C, Rabenau HF, Schwebke I. Joint communication from VAH and the Virus Disinfection Commission of the DVV and GfV: Wirksamkeit von Desinfektionsmitteln gegen Affenpockenviren. Status 2022 Jul 18. HygMed. 2022;47(7-8):158-64. Available from: https://vah-online.de/de/ and https://g-f-v.org/komissionen/

## Figures and Tables

**Table 1:**
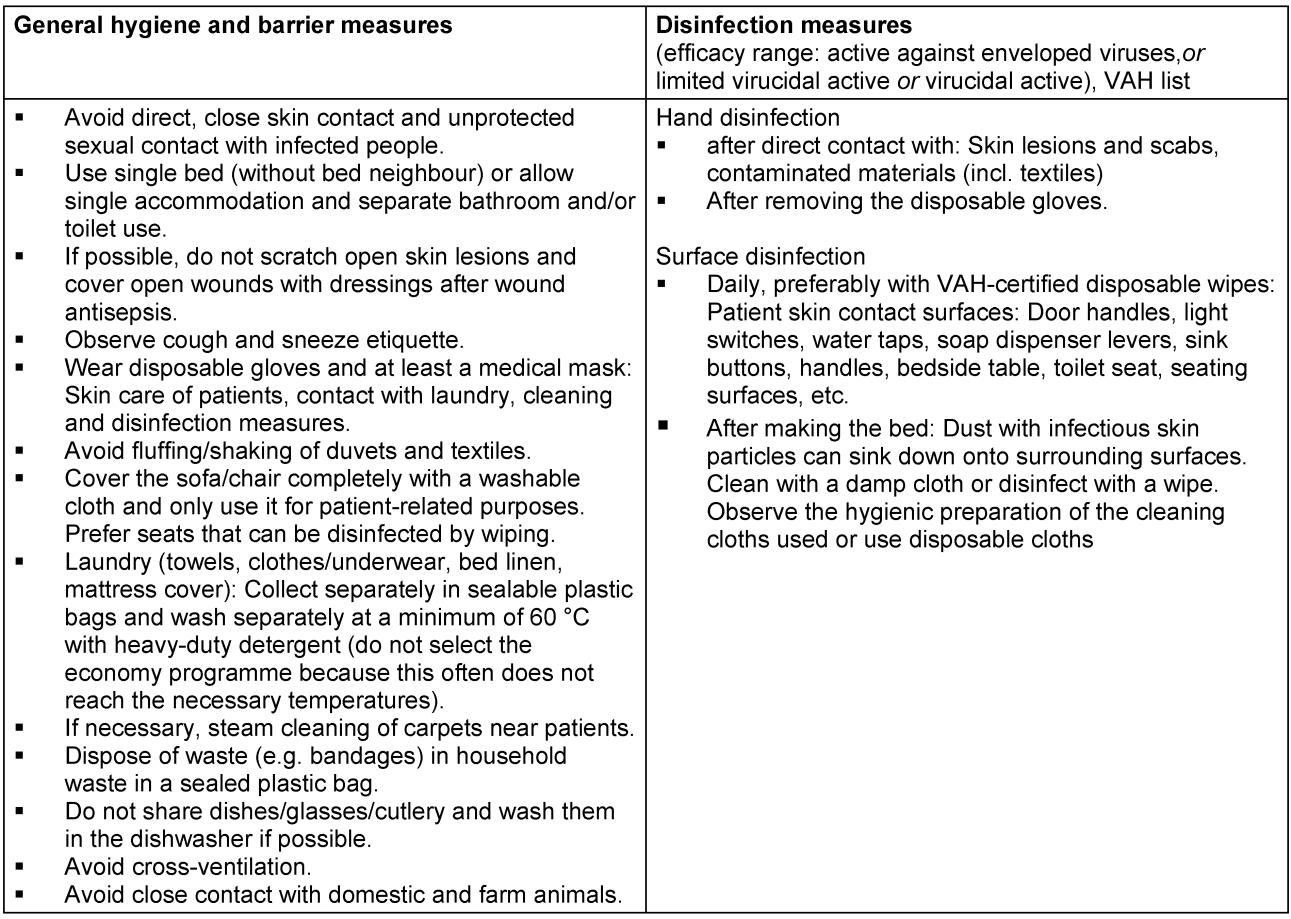
Hygiene measures and disinfection for households in which infected people live

**Table 2:**
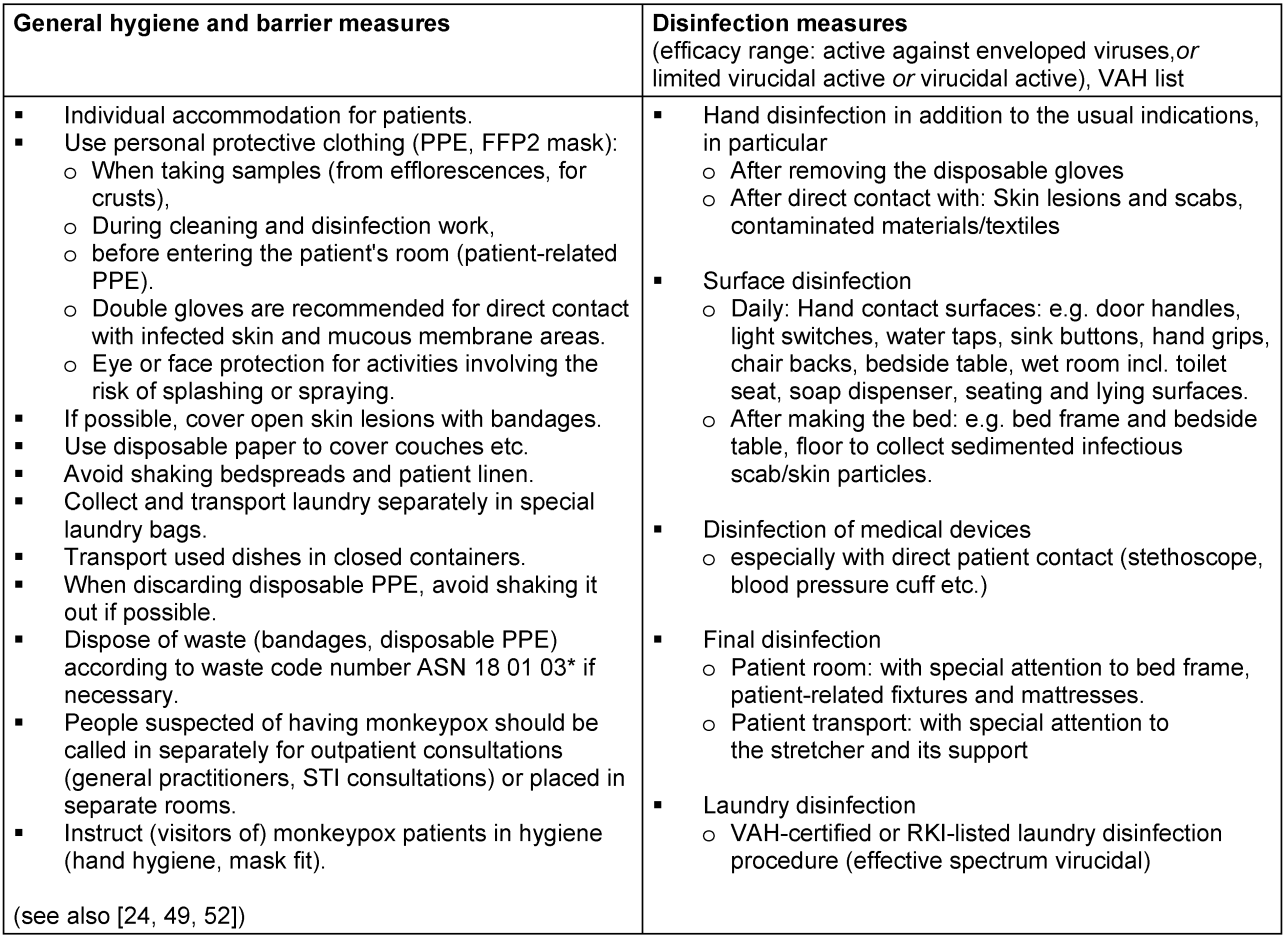
Hygiene measures and disinfection in the medical environment
